# Incidence and root causes of cancellations for elective orthopaedic procedures: a single center experience of 17,625 consecutive cases

**DOI:** 10.1186/1754-9493-8-24

**Published:** 2014-06-02

**Authors:** Ulla Caesar, Jon Karlsson, Lars-Eric Olsson, Kristian Samuelsson, Elisabeth Hansson-Olofsson

**Affiliations:** 1 Department of Orthopaedics, Institute of Clinical Sciences, Sahlgrenska Academy University of Gothenburg, Sahlgrenska University Hospital, SE- 413 45 Gothenburg, Sweden; 2 Institute of Health and Care Sciences Sahlgrenska Academy, University of Gothenburg, Gothenburg, Sweden

**Keywords:** Appointments and schedules, Operating rooms/organisation and administration, Waiting lists, Cancellation, Orthopaedic surgery, Perioperative nursing

## Abstract

**Background:**

The purpose of the Swedish public health-care system is to provide care on equal terms for all citizens. In this, as in most other systems where taxes and/or insurances pay for most of the care, normal market forces are set aside at least in part. At times, this has, for example, resulted in long waiting lists, particularly in terms of elective orthopaedic surgery, with several negative consequences, such as cancellations of planned surgery.

**Methods:**

The main purpose of this retrospective observational single center study was to evaluate and describe the number and reasons for cancellations in elective orthopaedic surgery. Studied were all the elective patients scheduled for joint replacement, arthroscopy and foot & ankle surgery, January 1, 2007 to December 31, 2011, whose procedure was cancelled at least once.

**Results:**

Of all 17,625 patients scheduled for elective surgery 6,911 (39%) received at least one, some several cancellations. The most common reason for cancelling a planned surgery was different patient-related factors 3,293 (33%). Cancellations due to treatment guarantee legislation reached 2,885 (29%) and 1,181 (12%) of the cancellations were related to incomplete pre-operative preparation of the patients. Organisational reasons were the cause of approximately 869 (9%) of the cancellations.

**Conclusions:**

In this study of patients waiting for elective orthopaedic surgery 6,911(39%) had their surgical procedure cancelled at least once, some several times. It appears that it should be possible to eliminate many of these cancellations, while others are unavoidable or caused by factors outside the responsibility of the individual clinic or even hospital. One possible way of influencing the high rate of cancellations might be to change the view of the patients and involve them in the overall planning of the care process.

## Background

More than 80% of Swedish health care is publicly funded with the aim of providing health care on equal terms to all citizens. In this, as in most other systems where taxes and/or insurance pay for most of the care, normal market forces are set aside at least in part. As a result, the demand for health care has a tendency to exceed the supply, resulting in waiting lists, among other things [1].

Elective orthopaedic surgery is one of the specialities where long waiting lists have become a well-known phenomenon [2-4]. There are several factors that are contributing to the accumulation of patients on waiting lists. One of the main reasons is that orthopaedic injuries and diseases that lead to surgery have increased markedly during the last few decades [5]. The increasing demand for surgical services has been caused to a large extent by an ageing population, better knowledge and new technical opportunities. These reasons indicate that even higher burdens will be imposed on the orthopaedic community in the near future [6].

These waiting lists comprise large numbers of patients and the effects include being unable to perform all the surgeries in a reasonable time, which leads to re-scheduling and cancellations. This causes a variety of problems for the patients and is also a major administrative logistical problem for the health-care system. In 2010, to reduce waiting times, the Swedish government introduced a law called the Treatment Guarantee, ensuring that the waiting time for elective surgical procedures should not exceed 90 days [7].

There are several well-known reasons for cancelling elective orthopaedic surgery. They range from an emergency case that must be prioritised to cancellation on the patient’s own request [8,9]. Other reasons are insufficient planning of the waiting list and the daily surgery schedule [10] resulting in cancellations that inevitably lead to extended waiting for the patients.

The prolonged waiting time is likely to cause unnecessary suffering and pain and possibly a deterioration in patient health, which might in turn cause delayed or impaired recovery and potentially a less favourable outcome [11,12]. In addition to these effects, cancellation or undue waiting correlates to a strong negative psychological concern for the patients [13-17].

Increased knowledge of the extent of and reasons for cancellations is needed to make the health-care service better equipped to manage and understand the patient’ needs when a cancellation occurs [14]. The aim of this study was to describe and analyse the number of and reasons for cancelling patients’ scheduled orthopaedic surgical procedures at a clinic treating both elective and acute patients.

## Methods

**Figure 1:**
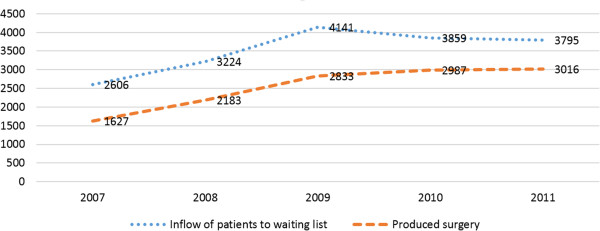
The inflow of all new patients to the waiting list and the produced surgery, 2007–2011.

The study was a descriptive single center study with retrospective observational data collection through the hospital’s records and registers. The study population comprised all the patients scheduled for orthopaedic surgery between, January 1, 2007 to December 31, 2011 at a University hospital clinic with an annual production of around 12,000 planned and acute surgical procedures. The orthopaedic clinic was organised into specialised teams focusing on trauma, joint replacement, arthroscopy, paediatric orthopaedics, foot & ankle, tumour and spine surgery.The patients included in the study were only those scheduled for joint replacement, arthroscopy, or foot & ankle surgery and then cancelled. The collected data included age, gender, diagnosis, reason for cancellation, time of cancellation and length of time until new scheduled surgery was performed. The age of the patients ranged between 13 and 99 years and 56% were women. The total inflow to the surgical waiting list of the selected patients during the study period was 17,625 persons. Of these, 12,646 (72%) underwent surgery at the current clinic, while the rest were either transferred to other clinics or definitely cancelled. This resulted in a difference between the inflow of patients and actual produced surgical procedures (Figure 1).

Definitions of certain terms: *Inflow of patients*; every new patient is entered into the electronic operation planning system as a file with a unique patient ID (waiting list). The patient remains in the planning system until the operation is completed, transferred to another caregiver or definitely cancelled. *Definitely cancelled*; the patient does not undergo surgery. *Transferred*; all planned procedures that the clinic was unable to perform within the three months’ treatment guarantee were cancelled at the current clinic and the patients were transferred to other care-givers. *Produced surgery*; all the patients who underwent surgery at the current clinic.

The scheduling of surgery was based on priorities and decisions made by the surgeons in consensus with the patients. A coordinator then booked the appointment for the surgical procedure, which means that patient data were entered into the planning system and a file with a patient ID was opened in the electronic planning system (Operätt). In this system, data were continuously registered by coordinators, surgeons and nurses. A special IT tool, Qlick View (QV), was used as a database and made it possible to identify, calculate and present quality measurements of all activities involving inflowing patients. Qlick View also made it possible to identify all cancellations made in the planning system. The planning system was validated every month. Cancellations were entered into the planning system under one of 61 possible codes. In order to make the results in the present study more comprehensible, these codes were merged into eleven categories (Table 1). The Swedish Ethics Committee, Gothenburg, approved the study, Dnr: 531–12. The data were managed using IBM SPSS Statistics (Version 21). Descriptive data is presented in absolute and relative numbers, median and range values. Graphics were illustrated using Microsoft Excel (Version 2013).

**Table 1:** Reasons for cancellations 2007-2011

	**2007**		**2008**		**2009**		**2010**		**2011**		**Total**	
Reason*	**Sum**	**%**	**Sum**	**%**	**Sum**	**%**	**Sum**	**%**	**Sum**	**%**	**Sum**	**%**
**1. Planned surgery was transferred**	614	6.2	429	4.3	430	4.4	859	8.7	553	5.6	2885	29.3
**2. Patient refrained from surgery**	350	3.5	338	3.4	279	2.8	356	3.6	349	3.5	1672	17
**3. Patient refrained from surgery for social reasons**	254	2.5	381	3.8	308	3.1	353	3.6	325	3.3	1621	16.5
**4. Incomplete pre-operative preparations**	154	1.5	289	2.9	205	2.1	282	2	251	2.6	1181	12
**5. Changes to the scheduled surgical programme**	148	1.5	109	1.1	136	1.4	314	3.2	162	1.6	869	8.8
**6. On-going infection**	157	1.6	144	1.5	170	1.7	114	1.2	101	1	686	7
**7. Medical reasons**	52	0.5	95	1	84	0.9	116	1.2	142	1.4	488	5
**8. Lack of personnel**	56	0.6	37	0.4	35	0.4	74	0.8	53	0.5	255	2.6
**9. Patient deceased or pregnant**	19	0.2	16	0.2	17	0.2	32	0.3	11	0.1	95	1
**10. Missing equipment**	6	0.1	4	0	4	0	16	0.2	15	0.1	43	0.4
**11. Lack of ward space**	4	0	9	0.1	24	0.2	1	0	0	0	38	0.4
	**1814**	**18.4**	**1851**	**18.8**	**1692**	**17.2**	**2517**	**25.6**	**1962**	**19.9**	**9836**	**100**

*Reasons for cancellation.

1. The planned surgery was transferred to another caregiver.

2. The patient refrained from surgery at the clinic, chose another hospital or abstained from surgery.

3. The patient refrained from surgery for social reasons.

4. Incomplete pre-operative preparations.

5. Changes to the scheduled surgical programme.

6. On-going infection(s) on the ward or the patient personally.

7. Medical reasons.

8. Lack of personnel.

9. The patient deceased or pregnant.

10. Missing equipment.

11. Lack of ward space.

## Results

**Figure 2:**
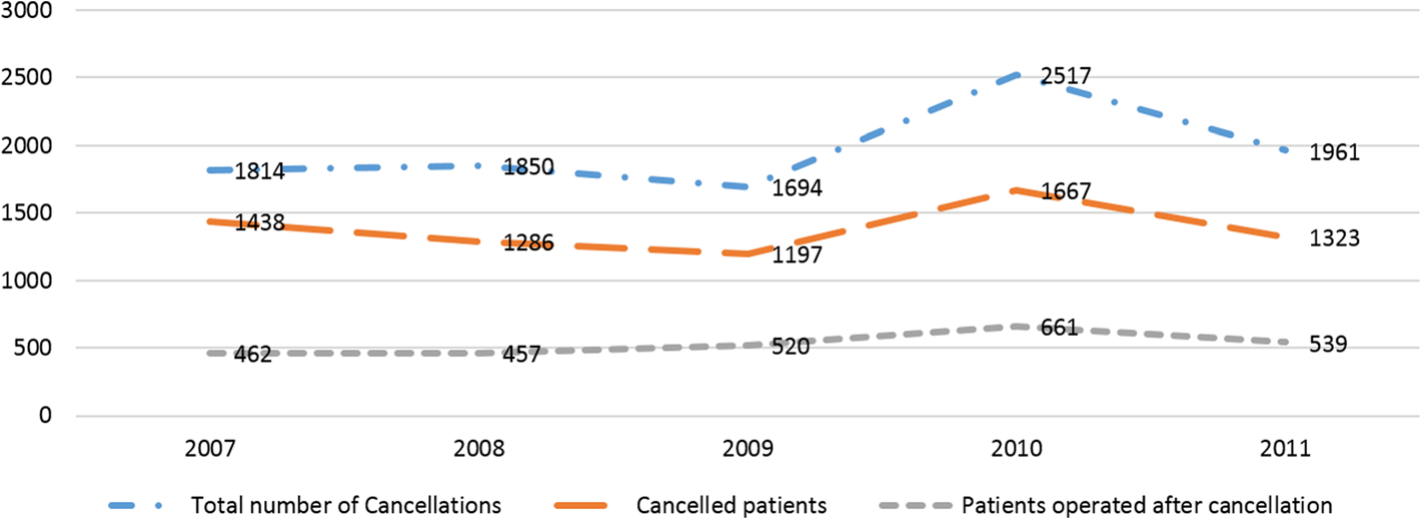
The number of cancellations, cancelled patients and patients operated on after cancellation (s) at current clinic.

Of all 17,625 patients scheduled for elective surgery, 6,911 (39%) had their procedure cancelled at least once. A quantity of 4,008 (58%) had their procedure cancelled once, 1,935 (28%) twice, 622 (9%) three times, 208 (3%) four times and 138 (2%) more than four times. This adds up to a total number of 9,836 cancellations for the 6,911 actual patients (Figure 2). Of these patients, 2,639 (38%) underwent surgery on a later occasion at the current hospital, while 4,272 (62%) were transferred to other clinics or declined surgery.

### Reasons for cancellations

Of all cancellations, 3,293 (33%) were based on the patients’ own requests. Almost half those cancellations 1,672 (17%) were due to the patients’ own wish to have the surgery performed on a later occasion, a definite cancellation or patients being unable to wait and for that reason choosing other alternatives. Cancellations related to family reasons, work or other social reasons were responsible for the remaining 1,621 (16%). More than 2,885 (29%) were due to the treatment guarantee and the clinic being forced to transfer patients to other care-givers, while 1,181 (12%) of the cancellations were due to insufficient medical assessment and/or arrangements related to pre-operative fasting or a lack of skin preparations in patients. Reasons related to the hospital’s organisation, such as incoming acute surgical cases replacing the planned procedures, the unexpected prolongation of on-going surgery and the delayed start of the planned procedure, were responsible for 869 (9%) of all the cancellations. Cancellations due to various infectious diseases were responsible for 686 (7%) and 488 (5%) of the cancellations were due to disorders that made the patient inappropriate at the planned time point. A shortage of nurses or physicians caused almost 255 (3%) of the cancellations, while 95 (~1%) was caused by other patient-related factors such as pregnancy. Approximately 81 (~1%) of the patients were cancelled due to a lack of important medical equipment or a lack of beds on the wards (Table 1).

### Cancellation time until surgery

In 671 (10%) of the 6,911 patients, the cancellation was decided less than 24 hours prior to the scheduled surgery. Of these same-day cancellations, 195(3%) patients were scheduled for a joint replacement, 417(6%) for arthroscopy of the knee, while 148 (2%) were scheduled for foot & ankle surgery.

### Time between cancellation and performed surgery

For those 2,639 (38%) patients who had their surgery performed at the current hospital after one or more cancellations, the waiting time for the re-scheduled surgery ranged between 0 and 1,896 days. Zero indicates that some had the re-scheduled surgery performed later on the same day the cancellation took place. The median waiting time ranged from 54 days for those who had been cancelled once to 96 days for those cancelled four times (Table 2).

**Table 2:** Median waiting time between cancellation and surgery

**Number of cancellations**	**1**	**2**	**3**	**4**
**Range, days**	0-1896	1-1713	1-1862	4-714
**Median, days**	54	56	67	96

### Cancellations and type of surgery

From the 6,911 cancelled patients, 2,833 (41%) came from the joint replacement team, 2,695 (39%) came from the arthroscopic team and 1,382 (20%) came from the foot & ankle team. The waiting time for the re-scheduled surgery varied between the different diagnostic groups (Table 3). No obvious reasons for these differences were found.

**Table 3:** Waiting time (days) for surgery after cancellation in the diagnostic groups frequently cancelled

	**0-7 days**	**8-30 days**	**31-90 days**	**> 90 days**
**Partial resection of the meniscus**	19	70	78	71
**Primary total knee replacement**	10	33	41	137
**Anterior cruciate ligament reconstruction**	5	38	66	58
**Primary total hip replacement**	16	25	41	78
**Arthroscopy other than meniscus resection**	12	38	61	34
**Acromioplasty (shoulder)**	3	10	28	25
**Major foot surgery**	2	10	13	22

## Discussion

The most important finding in the present study was the high frequency of planned orthopaedic surgery cancellations. Of all patients who had a scheduled time for planned surgery 6,911 (39%) had their surgical procedure cancelled at least once and some several times. In Sweden, the large university hospitals tend to have the longest waiting times for orthopaedic procedures [7]. Whatever the reasons for the long waiting lists, they lead to issues for the patients and organisational problems for the clinics, with overloaded surgical schedules and, at the end of the day, cancellations [18].

A delay in the planned surgery due to cancellation might contribute to the unnecessary “de-conditioning” of a pain-ridden patient and might also reduce the chances of achieving optimal results and/or prolong the postoperative rehabilitation process. The cancellation may also lead to a loss of confidence in the hospital, contributing to feelings of insecurity and uncertainty and thereby contributing to new cancellations. Last-minute cancellations have been shown to increase the patient’s fear and create a low level of trust in the hospital. It has also been shown that feelings of insecurity can lead to increased pain, leading to a prolonged hospital stay [14,15].

The present study revealed a variety of reasons for the cancellations. When categorised, one third could be attributed to the most common reason, i.e. patient-related 3,293(33%) (Table 1). The cancellations which took place on the patient’s own request, in order to have the surgery performed on a later occasion or because the patients could not manage to wait and therefore chose other alternatives, provided 1,672 (17%). The categories comprising family reasons, work or other social reasons were the causes in 1,621 (16%) of the cancellations. It is probably possible to avoid most of these cancellations. The category comprising patients who refrained from surgery at the clinic, chose another hospital or abstained from surgery also includes those who, during the waiting period, improved to such an extent that they abstained from surgery (Table 1). A careful examination and an improved dialogue with a deeper understanding on both sides, i.e. the patient’s and the nurse’s or physician’s, are likely to eliminate many of the cancellations in this category. It has already been suggested that improved patient information and education, as well as the more careful establishment of the indications for surgery, “might reduce the circumstances when surgery is no longer necessary” [19-22].

It is likely that, when patients feel that they are more involved in their care and know what will happen next, fear and doubt, contributing to cancellations, can be reduced. In a Norwegian study, the focus was changed to a new pathway where the patients themselves selected the day and time of surgery [23]. The hospital made a phone call to the patient two days before surgery to check that nothing was going to prevent the patients attending at the planned time. The hospital’s continuity resulted in a dialogue in which the patient could ask questions and the hospital could respond and support. This led to a high satisfaction rate for both parties and fewer cancellations compared with the group of patients that were treated according to the traditional pathway [24]. It has also been shown that patients’ wishes to know where they are on the waiting list and how they are prioritised are important if the patients are to feel involved and have the opportunity to control their own situation, which leads to more trust in the hospital’s planning systems and care [15]. Similar results have been reported when the concept of person-centred care (PCC) has been employed. In PCC, the patient is seen as an active partner involved in all decisions relating to the planning of his/her own care [25]. The concept of the patients being integrated into healthcare may decrease disappointments or unrealistic outcomes related to misunderstandings or miscommunication [26] Studies of PCC have reported positive outcomes for patients with hip fractures, as well as patients with heart failure, resulting in more involved patients and also in shorter hospital stays [27,28]. It has recently been found that the Swedish health-care system often fails to anticipate and respond to patients as individuals with particular needs, values and preferences [29].

Changing the view of the patient and including him/her in the whole planning process might be a way to reduce several of the reasons for cancellations, especially those directly related to the patient or to a poor pre-operative investigation [30].

In the present study cancellations related to incomplete preparations before surgery remained 1,181 (12%) (Table 1). Nurse-led pre-operative consulting has been shown to reduce the number of short-term cancellations, making the patients better informed, feeling safer and more motivated [30] the same findings as in the PCC concept. When the patients are well prepared before the operation, the cancellations for patient-related reasons decrease, resulting in fewer cancellations initiated by anaesthetists or surgeons on the day of planned surgery as well [19,24,31-34]. Pre-operative instructions not being followed or patients not being instructed adequately are issues that can be improved in order to reduce cancellations [19,35,36]. Having control of the situation and knowing what is going to happen next have also been shown to reduce the number of patients’ short-term cancellations [15].

In this study, 138 (2%) of the patients never showed up at the booked appointment for surgery. Sending a reminder text message a few days before the surgery to confirm the scheduled time has been shown to reduce the number of last-minute cancellations and also reduce the group of patients who do not show up at the appointed time [34,37].

The second most common cause of cancellations was the treatment guarantee, enforced in order to minimise or eliminate waiting times longer than three months. When a planned surgical procedure cannot be performed within the stipulated three months, the hospital has the option of transferring the patient to other care-givers. If the guarantee cannot be upheld, the hospital misses out on government money allocated for this purpose. The frequent use of transferring patients underscores the discrepancies between the demands for orthopaedic surgery and the clinic’s inability to satisfy these demands within 90 days. Even if the guarantee means that the patient will have surgery at an earlier time, it is still a cancellation and the patient is withdrawn from the waiting list and forced to have surgery elsewhere. It is likely that every cancellation, irrespective of its cause, is a disturbance that can have a number of varying, mostly negative consequences for the patients and the clinic. Waiting times for surgery cannot be regarded simply as an isolated phenomenon, they must also be considered in the wider perspective of the entire health-care system, at least in a specific area or county [38].

The present study can also be interpreted as a report on the situation when both elective and emergency cases are mixed at the same surgical clinic. Most of the research related to cancellations and waiting lists has focused on the planning and scheduling of elective surgery, although a common reason for cancelling elective surgery is prioritised emergencies. This study showed that almost 869 (9%) of all cancellations were due to emergency cases with higher priority [8,39,40]. The almost doubled number of cancellations in 2010 (Table 1, #5) most probably due to an extreme winter with months of icy and snowy streets can serve as an example. An overloaded surgical schedule might be avoided using an operating room reserved for emergency cases only. A separate orthopaedic trauma operation room has resulted in measurable changes, such as more emergency surgery being performed during the day, instead of the evening and during the night. It has also resulted in fewer complications due to a good fit with surgeons’ schedules and, as a result, less stress. The planned surgery was performed on time to a greater extent and cancellation rates dropped [41]. A reduction in emergency cancellations was related to fewer conflicts between elective and emergency surgery when the planned patients had a new pathway that reduced the rates of cancellations of elective surgery [23].

Allocating a special person responsible for the constant update of the emergency list has been shown to prevent over-optimistic surgical schedules and to have a beneficial effect on reducing the number of cancellations [42]. Having a visible whiteboard in the surgical clinic listing the acute patients waiting for surgery makes the staff aware and able to improve the scheduling and reduce cancellations [42]. In the United Kingdom, there are national guidelines recommending that hospitals with acute orthopaedic surgery should have a separate waiting list for trauma surgery which should be updated every day by a person in charge of the operating clinic. The separate waiting list helps the acute patients to undergo surgery on time, reduces the waiting stage and leads to a reduced number of cancellations [43].

Planning ahead in multi-professional teams in terms of available personnel resources, ward space limitations and necessary equipment has also been suggested to avoid several cancellations [44]. In this study, almost 336 (3.5%) were due to a lack of personnel, ward space or missing equipment.

It is obvious that some cancellations are unavoidable, such as when patients die, or the appearance of a new disorder that makes surgery at the planned time point inappropriate, an outbreak of contagion and so on. In the same way, it is obvious that the vast majority of cancellations can be prevented by improvements to the organisation [35].

### Limitations of the study

Since there is both a continuous inflow and outflow from the waiting list, the numbers given can vary. This makes it difficult to provide the precise numbers from one moment to another.

Another limitation could be that different staff categories entered the data into the surgical planning system. They might have had different views of using terms and knowledge when handling the computer-based system. This in turn could have led to inconsistent grouping and categorising of the reasons for cancellations.

This study showed the cancellations at one specific clinic only, making the reproducibility unproven.

## Conclusions

The number of cancellations of planned orthopaedic operations was high, of all 17,625 patients scheduled for elective surgery, 6,911 (39%) were cancelled at least once.

Many of the cancellations appear to be possible to reduce or eliminate, while others are unavoidable or might be caused by factors that are outside the responsibility of the individual clinic or even hospital.

By clarifying the reasons for the cancellations, everyone involved has better knowledge to improve and develop better routines to reduce the number of cancelled patients.

One way of influencing the high rate of cancellations might be to change the view of the patients and involve them in the overall planning of the care process.

The high number of cancellations in this study is a major quality problem affecting the individual patient and the actual health care organisation.

It is likely that cancellations are frequent also in other specialities.

## Competing interests

There are no financial competing interests.

## Authors’ contributions

UC EHO, JK were responsible for the study conception and design and the drafting of the manuscript, UC performed the data collection and UC EHO, performed the data analysis. UC EHO provide the ethical appropriate to the ethics committee. KS programmed the database EHO JK LEO KS made critical revisions to the paper. EHO supervised the study. All authors read and approved the final manuscript.
